# The Crustacean Hyperglycemic Hormone Superfamily: Progress Made in the Past Decade

**DOI:** 10.3389/fendo.2020.578958

**Published:** 2020-10-01

**Authors:** Hsiang-Yin Chen, Jean-Yves Toullec, Chi-Ying Lee

**Affiliations:** ^1^Department of Aquaculture, National Penghu University of Science and Technology, Magong, Taiwan; ^2^Sorbonne Université, Faculté des Sciences, CNRS, UMR 7144, Adaptation et Diversité en Milieu Marin, Station Biologique de Roscoff, Roscoff, France; ^3^Graduate Program of Biotechnology and Department of Biology, National Changhua University of Education, Changhua, Taiwan

**Keywords:** crustacean hyperglycemic hormone superfamily, structure diversity and evolution, biological functions, peptide structure, signaling pathway and receptor, Ecdysozoa

## Abstract

Early studies recognizing the importance of the decapod eyestalk in the endocrine regulation of crustacean physiology—molting, metabolism, reproduction, osmotic balance, etc.—helped found the field of crustacean endocrinology. Characterization of putative factors in the eyestalk using distinct functional bioassays ultimately led to the discovery of a group of structurally related and functionally diverse neuropeptides, crustacean hyperglycemic hormone (CHH), molt-inhibiting hormone (MIH), gonad-inhibiting hormone (GIH) or vitellogenesis-inhibiting hormone (VIH), and mandibular organ-inhibiting hormone (MOIH). These peptides, along with the first insect member (ion transport peptide, ITP), constitute the original arthropod members of the crustacean hyperglycemic hormone (CHH) superfamily. The presence of genes encoding the CHH-superfamily peptides across representative ecdysozoan taxa has been established. The objective of this review is to, aside from providing a general framework, highlight the progress made during the past decade or so. The progress includes the widespread identification of the CHH-superfamily peptides, in particular in non-crustaceans, which has reshaped the phylogenetic profile of the superfamily. Novel functions have been attributed to some of the newly identified members, providing exceptional opportunities for understanding the structure-function relationships of these peptides. Functional studies are challenging, especially for the peptides of crustacean and insect species, where they are widely expressed in various tissues and usually pleiotropic. Progress has been made in deciphering the roles of CHH, ITP, and their alternatively spliced counterparts (CHH-L, ITP-L) in the regulation of metabolism and ionic/osmotic hemostasis under (eco)physiological, developmental, or pathological contexts, and of MIH in the stimulation of ovarian maturation, which implicates it as a regulator for coordinating growth (molt) and reproduction. In addition, experimental elucidation of the steric structure and structure-function relationships have given better understanding of the structural basis of the functional diversification and overlapping among these peptides. Finally, an important finding was the first-ever identification of the receptors for this superfamily of peptides, specifically the receptors for ITPs of the silkworm, which will surely give great impetus to the functional study of these peptides for years to come. Studies regarding recent progress are presented and synthesized, and prospective developments remarked upon.

## Introduction

It is generally considered that crustacean endocrinology began in the 1920s when a series of studies were published (1–4) confirming that color changes in crustaceans are under hormonal control, although some earlier observations (5–7) had already revealed evidence of endocrine regulation in molting, coloration, and secondary sex characteristics [(8); see (9) for a detailed historical account]. The following years witnessed significant developments indicating that many other physiological processes are also under hormonal regulation by eyestalk-derived factors. Thus, inferences made mainly through ablation and replacement experiments confirmed the presence in the eyestalk of a wide array of presumptive hormones. Physiological processes suggested to be regulated by the eyestalk hormonal factors included, in addition to migration of chromatophoric and retinal pigments, carbohydrate metabolism, reproduction, molting and growth, osmotic and ionic balance, cardiac activity (10). A number of studies (11–16) helped determine the source of these hormonal substances in the eyestalk, the X-organ-sinus gland (XO-SG) complex. The hormonal factors are stored in the sinus gland (11, 17), a neurohemal organ (18), which lies next to a blood sinus (hence the name), into which the hormones are released upon stimulation. The sinus gland is a bulbous cluster of neurosecretory axonal terminals, of which the majority of the axonal input originates from the cell bodies of a cluster of neurosecretory cells (the medulla terminalis X-organ) where the hormones are synthesized [see (18)].

Strikingly, most of the presumptive factors have since been biochemically purified and characterized by bioassays, as functionally defined from the earlier studies. These hormones include a group of sequence-related and functionally diverse neuropeptides—crustacean hyperglycemic hormone (CHH), molt-inhibiting hormone (MIH), gonad-inhibiting hormone (GIH) or vitellogenesis-inhibiting hormone (VIH) (this hormone will be referred to as GIH in this review), and mandibular organ-inhibiting hormone (MOIH) that once constituted the entire CHH family (19–21). The peptides mainly consist of from 72 to more than 80 amino acids. The most remarkable characteristic is the presence of six particularly well-conserved cysteine residues forming three intra-molecular disulfide bridges, which has been shown to be an invariant signature for this superfamily, as it expands with the addition of new members. CHH from the shore crab *Carcinus maenas* is the first member peptide to be purified and its full amino acid sequence determined (22). This was followed by CHH isolated from the lobster *Homarus americanus*, which, interestingly enough, has both CHH and MIH activities (23), *C*. *maenas* MIH (24), CHH from the crayfish *Orconectes limosus* (19), and *H. americanus* GIH (25). Characterization of the sequences available revealed a new peptide family, with the realization that MIH and GIH are more similar to each other than each to CHH, in terms of sequence similarity, length, and modifications of the termini (MIH and GIH are free, whereas CHH is blocked, at both ends) (19, 20). MOIH was not characterized until a few years later when two biochemically MOIHs, differing from each other only by one amino acid, were purified and characterized from the crab, *Cancer pagurus*, with the sequences being more similar to MIHs than to CHHs (26). In the same year, three peptides from the spider crab *Libinia emarginata* were characterized and found to have MOIH as well as hyperglycemic activity (27). A cDNA encoding a CHH precursor protein demonstrated that the spider crab peptide with MOIH and CHH activities is indeed CHH as defined by sequence characteristics (28). The lobster CHH (23) and spider crab CHH (27), which respectively have MIH and MOIH activities, represent initial examples illustrating pleiotropy of these family members, which was frequently reiterated by later studies. The observations that some member peptides, in particular CHH, are pleiotropic and often overlap with biological activities of other members probably reflect the evolutionary history of the CHH family (29–31). Based on the structure of the genes and the hormone precursors, and the characteristics of the mature peptides, the CHH-family peptides were divided into two groups: Type I (CHH) and Type II (MIH/GIH/MOIH) (30, 32–34).

Increasingly, the CHH-family peptides have been identified more through nucleotide sequencing of transcripts (cDNA cloning or transcriptomics) than by the conventional approach of biochemical purification and peptide sequencing. In a recent report, *in silico* mining of transcriptome datasets from 112 crustacean species (representing three Classes: Malacostraca, Branchiopoda, Copepoda) resulted in a collection of 413 genes encoding CHH-family peptides (35). The CHH family has been expanded to the status of a superfamily as more member peptides are identified in non-crustacean species. The first non-crustacean member of the CHH-superfamily is the ion transport peptide (ITP), which was isolated from the corpora cardiaca of the desert locust *Schistocerca gregaria* and has structural characteristics that allow it to be integrated into the Type I group (36–38). The presence of the CHH-superfamily peptides is firmly established through *in silico* mining of data derived from several representative ecdysozoan clades [*e.g.*, (31, 39–42)]. Recently, the CHH superfamily has been expanded by addition of latrodectin peptides and HAND (helical arthropod-neuropeptide-derived) toxins present in the venoms of spiders and centipedes (43, 44). The fact that the CHH-superfamily peptides are expressed across the Ecdysozoa has singularly complicated our functional and phylogenetic understanding of the different members of the superfamily. Nevertheless, this diversity now gives timely opportunities to better understand the evolution of the CHH superfamily. Indeed, the grouping of the CHH-superfamily peptides has been revised according to analyses accommodating the newly discovered peptides, thus creating two new types: Type III and Type IV (31, 45). Investigation of the structure-function relationships of the peptides can now benefit as more structural variants with novel functions are available for comparative study, as exemplified by the structural study of HAND toxins (43).

The existence of multiple copies of genes encoding the crustacean members of the CHH superfamily increases the structural diversity of the member peptides, although the physiological significance of such structural polymorphism has not been fully clarified (29, 30). Alternative splicing of RNA also contributes to the structural diversity of the Type-I peptides (37, 46–55). Thus, at the mature peptide level, two peptides are derived from a common transcript, a short-splice form (CHH or ITP) and a long-splice form (CHH-L or ITP-L), which share the same sequence for the first 40 residues from the N-terminal end but differ considerably after the 40^th^ residue (56, 57). The peptides of the long-splice form have been far less characterized functionally than their short-splice counterparts. Recent studies utilizing RNA interference (RNAi), however, have shed some light on their functions (58–60). Structural diversity is further augmented by a post-translational isomerization of specific residues (Phe^3^ in CHH and Trp^4^ in GIH), resulting in a change in the configuration of the residue from L-form to D-form (61), which modifies the functionality of the peptides (62–66).

While the XO-SG complex in the eyestalk is the first tissue from which the crustacean members of the CHH superfamily were isolated and most likely the main source of these peptides, it has been shown that they are also expressed in other nervous and non-nervous tissues [*e.g.*, (47, 48, 50, 53, 55, 67–72)]. Similarly, ITP and ITP-L are widely expressed in the central and peripheral nervous system (49, 73, 74). The broad tissue expression of the CHH-superfamily clearly attests to their functional importance and might be closely related to the observed pleiotropy of these peptides. Physiologically adaptive roles of CHHs have been put to test using animal models that undergo life-history stages that presumably require regulation by CHH (75, 76). Recently, the metabolic effects of CHH on the muscle and hepatopancreas of the crayfish *Procambarus clarkii* were comprehensively characterized using a metabolomic analysis, revealing that the effects are more wide-ranging than previously realized and that the two tissues are differentially regulated by CHH (77, 78). The metabolic effects of CHH have also been implicated in the pathogenesis of diseases in infected crustaceans (77–81). On the other hand, several studies suggested that CHH modulates the immune functions (82–84), although the proposed immunomodulatory effects of CHH have not been thoroughly assessed in pathologically relevant conditions. It is entirely possible that an observed functional change in response to CHH treatment may be due directly to metabolic effects (especially energy metabolism) of CHH, as already suggested by several authors (70, 75, 85, 86). The fact that a given peptide member could be expressed in multiple tissues complicates physiological investigations. A case in point is the ecdysis-associated hemolymph surge of CHH that is released from gut endocrine cells, not the eyestalk XO-SG complex (68). The mode of action, endocrine or autocrine/paracrine, would be another issue that should attract the interest of physiologists working on the function of these peptides.

Structure-function relationships have been comprehensibly studied for MIH of *Marsupenaeus japonicus* (Pej-MIH), ITP of *S. gregaria* (Scg-ITP), and CHH of *Scylla olivacea* (Sco-CHH) using mutated recombinant peptides (87–90). Moreover, the three-dimensional structure of three crustacean member peptides (Pej-MIH, Pej-CHH-Gly, and Sco-CHHL) and that of a HAND toxin (Ta1a) are available for comparison (43, 91–93). Several functionally important residues, as assessed by the studies of the structure-function relationships are located in the parts of the structure that are in close proximity, which are likely involved in receptor binding and activation (87–90, 93). An exciting and important discovery in the field of study of the CHH superfamily is the identification of three orphan G protein-coupled receptors (GPCRs) as receptors for the silkworm *Bombyx mori* ITP and ITP-L (94), which subsequently led to identification of several crustacean GPCRs as the candidate receptors for crustacean member peptides of the superfamily, CHH or MIH (95–97). Identification of the receptors would give great impetus to many aspects of functional studies, including confirmation of target tissues, functional specificity (and overlap), and the cellular mechanism of action coupled to receptor activation.

This review is intended to highlight and discuss the progress that has been made in the past decade or so in the field of study of the CHH superfamily within the general framework of the knowledge accumulated since almost a century ago. Readers are also referred to reviews on similar topics that have been recently published (56, 98).

## Evolution and Structural Diversification Of The Superfamily

### Genomic Diversity

Isoforms of CHH were firstly characterized with regard to their specific functions. For example, CHHs *sensu stricto* are pleiotropic hormones mainly involved in the regulation of metabolism or water and ion balance, or may even have a negative effect on reproduction and molting. However, MIH/GIH/MOIH seemingly have, in the current state of our knowledge, more limited functions. These functional differences are obviously associated with structural differences which have enabled them to be distinguished and classified as distinct types, such as Type I and Type II, respectively (33). Type I brings together peptides with a cryptic sequence or PRP (precursor related peptide) and a dibasic cleavage site upstream of the mature peptide sequence. Type II is characterized by the absence of this PRP and has a glycine residue in position 5 after the first cysteine (33–35). It is important to keep in mind that if the first sequences were obtained from an identified biological function, this is no longer the case for most member peptides identified subsequently, as most have been characterized mainly by high-throughput sequencing techniques.

CHH-superfamily members are clearly no longer restricted to crustaceans, since ITP (ion transport peptide) was found in locusts (37, 38). ITP has since been found not only in other hexapods but also in chelicerates (39–42, 44, 99–101) and nematodes (102). ITPs were initially believed to be specific to non-crustaceans until they were identified in non-malacostraca crustaceans such as phyllopods (31, 103, 104), copepods (105–107), and remipedes (108). In these species, the presence of ITP seems to be exclusive since no other CHH peptide has been characterized simultaneously. This isoform presents a precursor organization closer to that of CHHs than MIH/GIH/MOIH, *i.e.*, the presence of a PRP and a dibasic cleavage site, which has led to its inclusion in Type I (31). However, phylogenetic studies, carried out on the basis of sequences of the three isoforms, clearly place the ITPs, including those obtained from non-malacostracan crustaceans and hexapods, at the base of Types I and II. This observation led to the proposal to create a third group, Type III, which brings together all these ITP orthologues (31, 45).

The analysis of recent transcriptomes has added another level of complexity by identifying a new form of peptide belonging to the superfamily (45, 109, 110). These new sequences possess the six conserved cysteine residues, but do not have a PRP sequence, nor a dibasic cleavage site or the glycine residue, a signature of MIH (45). They are also significantly longer than other isoforms with more than 80 amino acids. Nevertheless, their BLAST hit clearly clusters them with ITP isoforms of insects and Cladocera crustaceans such as *Daphnia* rather than CHHs or MIH/GIHs. For this reason, they have been grouped together under the name ITP-like (45). Thus, inclusion of ITP-like sequences in a phylogenetic analysis conducted on more than 200 sequences of family members confirms their belonging to the same group and positions them as an independent group (45). The features of their sequences exclude them from the types currently recognized and once again question the dichotomous classification proposed by Lacombe et al. (33). It constitutes a new set of members in the same way as CHHs *sensu stricto* or MIHs and would thus form a fourth type (Type IV), if we refer to phylogeny.

The analysis of the different transcriptomes also addresses the number of isoforms, notably CHHs, present within each species. Information collected from these analyses revealed the presence of many additional isoforms within each species, which are growing more numerous as the number of tissues analyzed has increased. While there are so far only one or two isoforms of MIH/GIH (111–116), CHH peptides exhibit much greater variety with commonly four or more different isoforms (45, 117). The presence of multiple *chh* genes has been demonstrated in decapods, (up to nine) whereas only one copy of the *itp* gene is present in insects (29–31, 57). Similarly, only two *mih* genes have been identified in several species (111, 118). Some are clearly derived from recent duplications and show little divergence (119), while others differ substantially from the most represented isoforms and clearly demonstrate that diversity has been underestimated (45). It is very likely that additional transcriptomic analyses will uncover new isoforms, even in species that have already been well studied (119). This diversity supports the importance of this molecular family at the physiological level and could also explain the apparent pleiotropy of the CHHs.

Beyond this genomic diversity, there are mechanisms in some crustaceans that can produce new isoforms by post-transcriptional or post-translational modification, as detailed below.

### Post-Transcriptional Modifications

**Figure 1:**
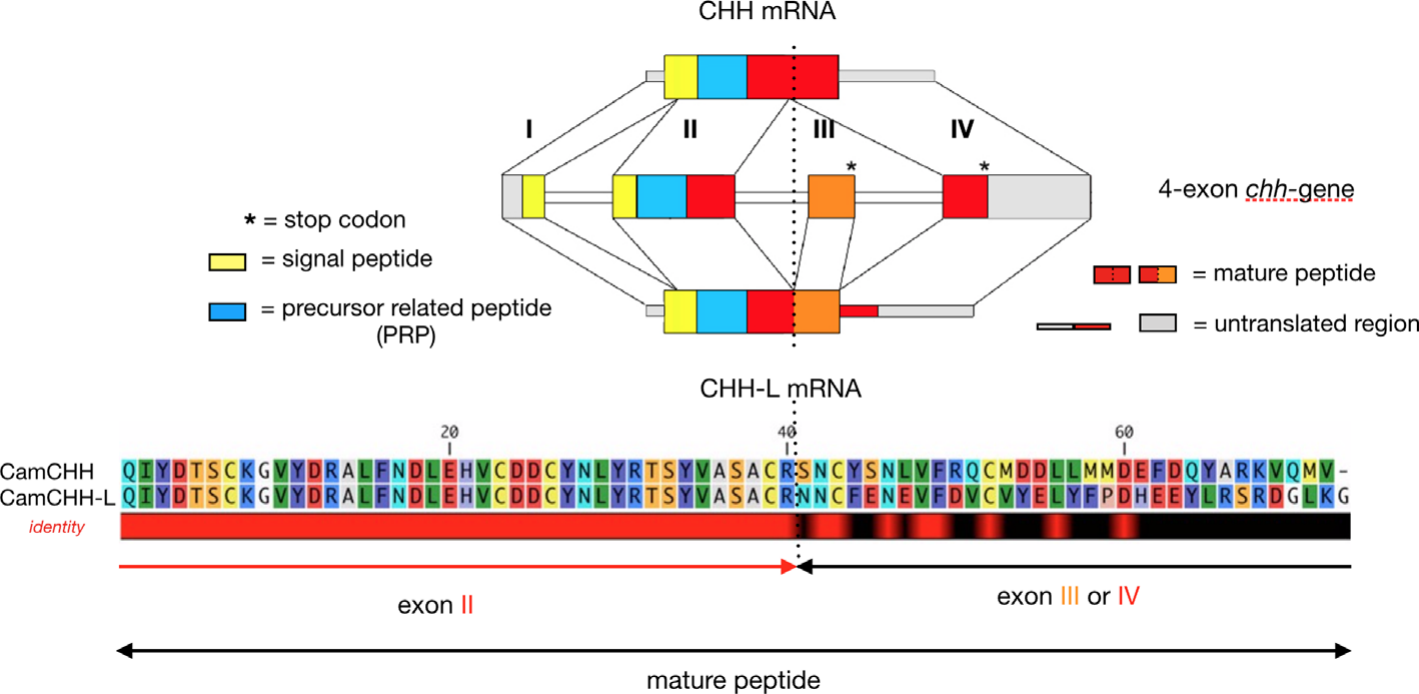
RNA alternative splicing of the *chh* gene in the shore crab *Carcinus maenas*. *C. maenas chh* gene is a 4-exon gene. The long-splice form (CHH-L mRNA) consists of exon I, II, III, and IV, and the short-splice form (CHH mRNA) consists of exon I, II, and IV. The first 2 exons (exon I and II), which encode the signal peptide, the precursor-related peptide (PRP), and the first 40 residues of the mature peptide, are common to the two splice forms. The remaining sequence of the CHH-L and CHH mature peptide is encoded by exon III and IV, respectively. Note that both exon III and IV have a stop codon. The dashed line marks the splice site.

Alternative splicing of the *chh* gene in pericardial organs of *C. maenas* (47) results in a CHH isoform that lacks the defining activity of CHH. Alternative splicing has since been demonstrated for several CHH genes in malacostracan crustaceans (47, 48, 53, 60, 72, 120) but also for insect ITPs (51, 52, 57, 59). These splicing patterns originate in the structure of the *chh* or *itp* genes, which have a minimum number of four exons. The splicing alternates two exons (exon 3 or 4) as the terminal part of the transcript, resulting in two transcript forms—the short-splice forms (exons 1, 2, 4) and the long-splice forms (exons 1, 2, 3, 4), in which the stop codon is located at the end of the third exon, leaving the fourth exon untranslated (**Figure 1**). This process generates two peptides from a single gene that differ in sequence after the 40^th^ amino acid residue of the mature peptide. Exons 3 and 4 both encode peptide sequences of similar lengths, with two cysteine residues in the same positions, thus suggesting that alternative exon 4 appeared by tandem duplication of exon 3, or vice versa (31). The transcripts of the short-splice forms of CHH peptides were mainly found in neurons of the central nervous system (more precisely in the X-organ located in the eyestalk), whereas the long-splice forms were found in neurons of the peripheral nervous system and non-nervous tissues, thoracic ganglia, Y-organ, mandibular organ or hemocyte according the species (47, 48, 55, 60, 70, 121). Peptides encoded by the long-splice forms of CHH and ITP transcripts are slightly longer than peptides encoded by the short-splice transcripts and are never C-terminally amidated [see (56)]. They are named CHH-L and ITP-L, respectively. In addition to these genes with four exons, there are also genes with only three or even two exons, such as the majority of CHH isoforms of penaeid shrimps. MIH and MOIH are encoded by genes with three exons (29, 31, 122, 123). Alternative splicing mechanisms, as described here, have never been reported for these two- or three-exon genes of penaeid shrimps. It is also interesting to note that this splicing process can generate “truncated” forms of CHH as demonstrated in the hemocyte of the crayfish *P. clarkii* (72), resulting in a precursor, where the mature peptide sequence is truncated with only the first 40 residues.

### Ubiquity of the CHH-Superfamily Peptides

The following examples underline the widespread ubiquity of the CHH superfamily members, which demonstrates the surprising diversity of these peptide isoforms and their functions. Indeed, if the first isoforms were characterized at the level of neural ganglia, first in the XO-SG complex in the eyestalks and then followed by the thoracic ganglia, the detection of these peptides in other tissues is becoming increasingly common. The first evidence was provided by immunocytochemical analysis in *C. maenas*, in cells located at the fore and hindgut level, suggesting a mechanoreceptive function. These are “paraneurones” which secrete CHH into the hemolymph during ecdysis (68, 124). Since then, it has been shown that CHH-related peptides are expressed in a wide variety of tissues including the pericardial organ (120, 125), retina (126), gill (60, 127), spermatophore (71, 128), Y-organ (121, 129), hemocyte (70, 72), ovary (129, 130), stomach (130), and intestine (54).

Until recently, it was thought that MIH/GIH/MOIH peptides were only expressed in neuronal tissues of the XO-SG system. However, MIH expression has been identified in the heart of the shrimp *Litopenaeus vannamei* (131), and in the gill, ovary, and abdominal ganglion of *Macrobrachium nipponense*, though at relatively lower level than in the eyestalks (132).

### Post-Translational Modifications

In parallel with gene multiplication or post-transcriptional processes such as alternative splicing, peptide modification in some taxa further increases the diversity of isoforms and functions of Type I or II members. This mechanism, hitherto restricted to Astacidea in crustaceans but present as well in other arthropods such as arachnids and mollusks and a few vertebrates, involves production of peptides in which the chiral form of a single amino acid has been modified, *i.e.* from the L-enantiomer to the D-enantiomer (61). Thus, lobsters and crayfish have CHH isomers that are differentiated by the configuration of the Phe^3^, as either an L-form or a D-form. Both isomers exhibit the defining hyperglycemic activities and regulate energy metabolism, but the change in configuration results in modification of the biological activity of the peptide (64, 66, 133). In particular, the hyperglycemic response kinetics are delayed with d-Phe^3^-CHH (3 to 4 h instead of 2 h for the L-isoform), and the amplitude of the hyperglycemic response is increased (10x higher for d-Phe^3^-CHH). Additional functional changes for d-Phe^3^-CHH were reported as having more potent inhibitory activity on the molting gland during ecdysteroidogenesis (65) or having a higher osmoregulatory activity (62, 63).

A similar modification from l-GIH to d-GIH has also been demonstrated in three Nephropidae species (American, European, and Norway lobsters). In this case, the modification is located at the level of the fourth N-terminal amino acid, a tryptophan (134, 135). However, the function of d-Trp^4^-GIH is still unknown, as it does not display significantly inhibitory effect in a heterologous *in vivo* assay as its counterpart does.

**Figure 2:**
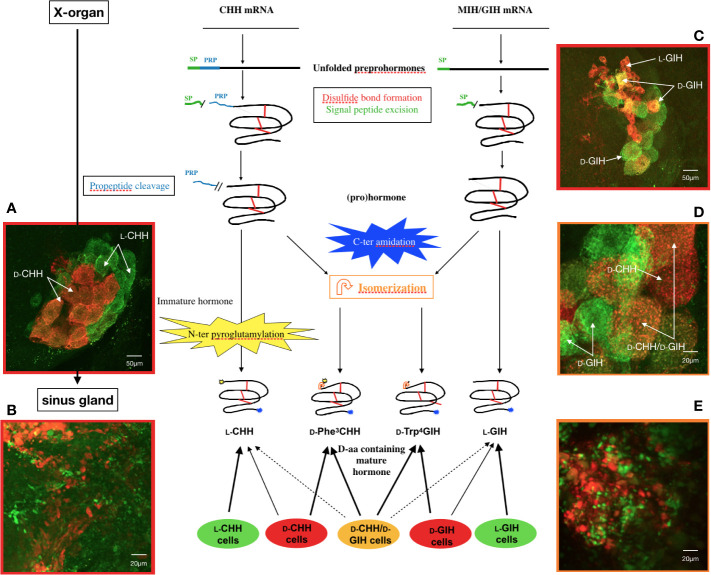
State of the current understanding of L- to D- isomerization in *Homarus americanus*. General diagram of precursor processing of CHH and GIH isomers in relation to the different cell types in the X-organ–sinus gland complex and confocal micrographs of double labeled whole mounts of lobster *Homarus americanus*. Amidation can occur pre-, co- or post-cleavage of PRP. Cyclization of the N-terminus of CHH is optional (*i.e.*, N-terminus unblocked CHH can be released) and, similarly to isomerization, it occurs after PRP cleavage. GIH is not N-terminally cyclized (by pGlu). SP, signal peptide; PRP, precursor-related peptide. **(A)** Distribution of CHH cells in the X-organ showing green somata (L-CHH cells) and orange somata (D-CHH cells). **(B)** Axon terminals in the sinus gland showing secretory granules in L-CHH cells (green) and D-CHH cells (red). **(C)** General view of the X-organ visualizing small L-GIH cells (red) and larger D-GIH cells (green or yellow). L-CHH and L-GIH cells secrete exclusively CHH and GIH, respectively, whereas D-CHH and D-GIH cells release mainly the D-isomer of the respective hormone, in addition to a variable amount of L-isomer. **(D)** Immunolocalization of D-Trp^4^ GIH and D-Phe^3^ CHH in the X-organ where three cell types were observed: D-CHH cells (red), D-GIH cells (green) and D-cells producing both D-isomers (orange). D-cells secrete mainly the D-form of both CHH and GIH. Besides isomerization, the same posttranslational processes occur in every type of CHH or GIH cell. **(E)** Sinus gland axonal arborizations containing D-Trp^4^ GIH (green) and D-Phe^3^ CHH (red) (135).

The study of the maturation dynamics of the CHH isomers highlighted the different stages of the post-translational processing of the precursor (**Figure 2**). It appears that isomerization takes place after cleavage of the pro-peptide and before N-terminal cyclization (136, 137). The production of antibodies specifically directed against the two CHH isomers made it possible to monitor *in situ* their location during the maturation process in the XO-SG complex of eyestalks of the crayfish *O. limosus* and the lobster *H. americanus* (135, 136). Two cell types producing either L-CHH exclusively (L-CHH cell) or a mixture of both L- and D-isomers (D-CHH cell) were identified (**Figure 2**). Most of the transformation of the L-isomers seems to occur in the cytoplasm, before the granules penetrate the axons (138). A similar approach was conducted with GIH isomers. Differential localization of the two isomers in different cells was characterized, as for CHHs. In the same way, simultaneous monitoring of the four isomers showed co-localizations within the same cells. Only L-GIH and L-CHH could not be found together (135) (**Figure 2**).

These studies have highlighted a particularly original mechanism of post-translational modification and curiously, so far, this has been demonstrated only in Astacidea crustaceans. Because this L-to D-post-translational modification is subtle and not detectable by most sequence determination approaches, it cannot be excluded that it exists in other taxa, although it has been searched for in other crustaceans, without success. Nevertheless, this process is not specific to crustaceans. As mentioned above, it has not only been found in arachnids, but also in molluscs, tetrapods, and mammals (61).

Another post-translational modification, a C-terminal amidation event, seems particularly important to limit degradation of the peptides by carboxypeptidases (139). The C-terminal amidation concerns only CHH and ITP, while the long-splice form (CHH-L or ITP-L) do not present this modification. In addition to the protective role in resisting degradation, the amidation is also important for the activity of the peptides, since the amidated peptides, compared to the un-amidated ones, have more potent activities (139–141). This post-translational modification has also been infrequently observed in type-II peptides such as GIH or MIH, although the functional significance of the modification is not clear (132, 142, 143). Like the C-terminal extremity, the N-terminus has, again only for CHH and ITP, a post-translational conversion *via* cyclization of the terminal Glu or Gln residue into pyroGlu (56, 57). This modification would protect the N-terminus from degradation by aminopeptidases, but does not significantly change the biological activities of the peptide, as the N-terminally unblocked and blocked CHHs of *C. maenas* were shown to be almost distinguishable in the activities of elevating hemolymph glucose levels and of repressing ecdysteroid synthesis (144).

### Weaponization of the CHH-Superfamily members?

Among the peptides characterized with D-amino acyl residues, it is interesting to note that there are many examples of these molecules that have been extracted from venoms or the nervous system (145, 146). This link between members of the CHH superfamily and venom peptides was further strengthened by the discovery that certain peptides of low molecular weight identified in black widow spider venom (genus *Latrodectus*), e.g., the latrodectins, show sequence similarities with CHH, particularly in terms of size, around 70 residues, the signature of the six conserved cysteines including similar disulfide bond pairing, and a similar alpha-helical structure (99). Further analyses suggested that CHH-superfamily peptides might have been recruited for venom expression at least three times: once in Hymenoptera, once in scorpions, and at least once in spiders, each lineage independently evolving venom production (44). However, the functional role of the latrodectins remains unclear as they are not insecticidal or toxic to mice (99), nor do they produce hyperglycemic effects when injected into crabs, crayfish, or shrimps (147).

**Figure 3:**
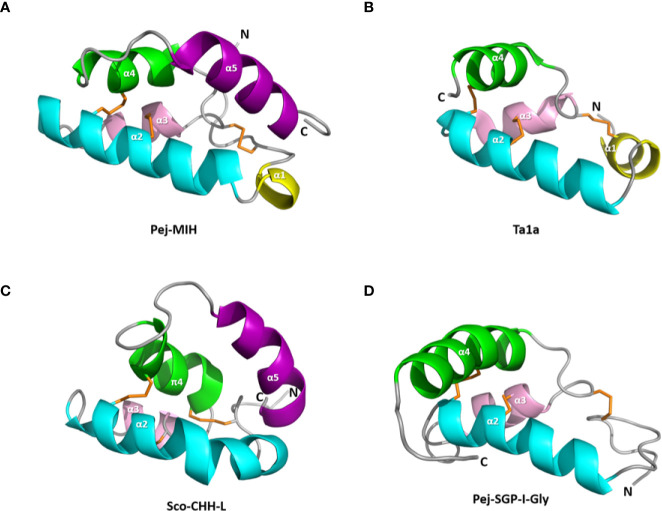
Structure of the spider toxin Ta1a and representative crustacean member peptides. Ribbon structure of **(A)** Pej-MIH, **(B)** Ta1a, **(C)** Sco-CHH-L, and **(D)** Pej-SGP-I-Gly. The helices, and N- and C-terminus (N and C) are labeled. Note the absence of the terminal helix (α5), which is present in both MIH and CHH-L, from Ta1a and Pej-SGP-I-Gly. A π helix (π4), topologically equivalent to the α4 in other structures, is present in Sco-CHH-L. Protein Data Bank IDs for the structures are respectively 1J0T, 2KSL, 5XS1, and 5B5I.

An exception was however first identified in the venom of common agelenid spider *Tegeneria agrestis*, the U1-agatoxin-Ta1a (148, 149), and then discovered in the venoms of members of the centipede genus *Scolopendra*, representing convergent recruitment of CHH-superfamily peptides into the toxin arsenals of these myriapods (43). Despite a weak sequence identity, the structure of MIH of the shrimp *M. japonicus* and CHH-L of the crab *S. olivacea* are surprisingly topologically similar to that of Ta1a. However, the N-terminal α-helix (α 5), present in both MIH and CHH-L, is absent from Ta1a [**Figure 3**; also see (43)] and the loss of this helix could be a key step in weaponization of CHH family peptides (43).

Recently, a proteomics approach of the venom components of the parasitoid wasp *Tetrastichus brontispae* resulted in the identification of, among a large number of diverse proteins or peptides, three ITP-L (ITPLn1-3) as abundant toxins (150). These ITP-Ls are distinct from ITPs by the implementation of a possible alternative splice allowing diversification of the gene products and a potential neo-functionalization that would lead to an evolution towards peptides of the CHHs family with venomous properties.

The mechanisms for recruiting members of the CHH superfamily as toxins are clearly varied and appear to have evolved independently in the different taxa. Nevertheless, the use of ITP/CHHs as evolutionary fodder for these transformations is a consensus among a growing number of species and is certainly not fortuitous. The structural properties (3 disulfide bridges which guarantee great stability), the multiplicity of genes and the post-transcriptional or even post-translational mechanisms are all processes that can lead to neo-functionalization, of which the acquisition of toxicity is one. In this context, one of the consequences of the acquisition of a D-amino acid peptide is a higher resistance to proteases and therefore a longer half-life for these peptides (146). In the future, it would not be surprising to find organisms with venomous ITP/CHHs carrying this type of modification.

## Biological Functions

### Type-I and III Peptides

CHH and ITP were functionally defined by the hyperglycemic activity and stimulation of trans-epithelial Cl^–^ transport in the ileum, respectively, which formed the basis (by bioassay) for purification, chemical characterization, and eventual identification of the hormones (19, 22, 37, 38, 151, 152). Studies over the years have however revealed that the two peptides are more functionally conserved than the initial defining functions would have suggested.

#### Metabolism, Ionic and Water Homeostasis, and Development (CHH, ITP, CHH-L, and ITP-L)

**Figure 4:**
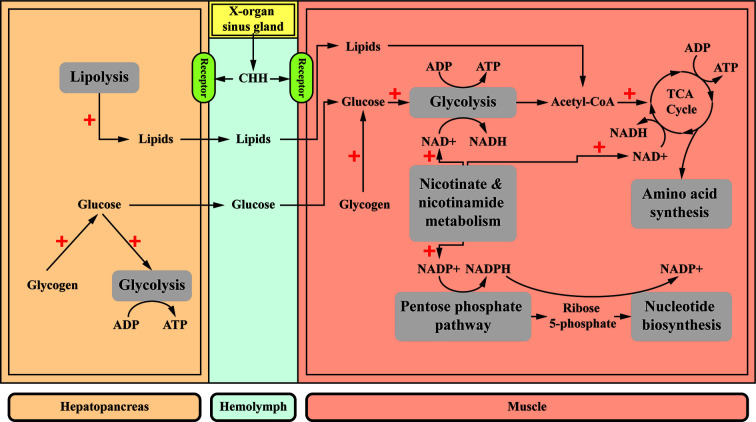
A proposed scheme for the metabolic roles of CHH in the muscle and hepatopancreas. CHH differentially regulates its target tissues. CHH decreases glycogen accumulation in both target tissues, resulting in higher levels of glucose (154–158) that drives glycolytic flux (160, 161). Moreover, in the hepatopancreas, CHH enhances lipolysis (77, 163). Glucose and lipids are released into the hemolymph and taken up by the muscle where they are further metabolized *via* glycolysis and TCA cycle, respectively, for ATP production. In the muscle, central to the effects of CHH is a stimulated “Nicotinate and nicotinamide metabolism”, which provides two nicotinamide coenzymes (NAD^+^ and NADP^+^) that drive glycolysis and TCA cycle, and the pentose phosphate pathway, respectively, resulting in increased ATP supply and biosynthesis of amino acids and nucleotides (77, 78). **+**, stimulatory effects.

A “diabetogenic factor”, as the presumptive factor elevated hemolymph glucose levels, in the crustacean eyestalks was suggested more than 7 decades ago (153). It is generally accepted, based on early studies, that CHH mobilizes glycogen reserves in the CHH target tissues (*e.g.*, the hepatopancreas and muscle), leading to hyperglycemia *via* regulation of the amount and activity of the enzymes (glycogen synthase and glycogen phosphorylase) involved in glycogen metabolism (154–159). The increased availability of glucose for cells, resulting from the glycogen-mobilizing effect of CHH, may subsequently stimulate the glycolytic flux (160, 161). The stimulatory effects of CHH on the release of amylase from the hepatopancreas (162) and hemolymph levels of triacylglycerols and phospholipids (163) have been reported but not further characterized. Recently, the metabolic roles of CHH in the crayfish *P. clarkii* were more comprehensively characterized using an RNAi approach (double-stranded RNA) followed by profiling the hepatopancreas and muscle metabolomes (77, 78). The combined data indicated that CHH has more diverse effects than previously realized, and the two target tissues are differentially regulated. The main effects of CHH are stimulation of glycolysis and lipolysis in the hepatopancreas, and higher rate of utilization of carbohydrates (glucose and other sugars, including fructose, galactose, sucrose, and lactose) *via* glycolysis and TCA cycle (resulting in higher levels of ATP), stimulation of the pentose phosphate pathway (PPP) flux (leading to increased levels of nucleotide biosynthesis), and elevation of amino acid biosynthesis in the muscle (77, 78). Stimulation of the “Nicotinate and nicotinamide metabolism”, which concerns the metabolism of two nicotinamide coenzymes (NAD^+^ and NADP^+^), is central to the metabolic effects of CHH in the muscle. Thus, the higher levels of NAD^+^ (and higher NAD^+^/NADH ratio) and NADP^+^, respectively, drive these fluxes through glycolysis, the TCA cycle, and the PPP (77, 78). The tissue-specificity of CHH regulation is consistent with the results showing the transcript expression of carbohydrate metabolism-related enzyme genes were differentially regulated by CHH in the same two tissues of *M. japonicus* (159). A proposed scheme for the metabolic roles of CHH in the two target tissues is shown in **Figure 4**, based on combined data from several related studies.

A substantial amount of evidence has been accumulated indicating that CHH is involved in regulating osmotic homeostasis. It was first reported in the American lobster *H. americanus* that sinus gland extracts increased the osmoregulatory capacity of eyestalk-ablated animals kept at low salinity (164). A CHH variant, specifically D-Phe^3^-CHH, when injected was able to significantly compensate the eyestalk ablation-induced decrease in hemolymph osmolality, Na^+^ concentration, or both, in *H. americanus* (164) and the freshwater crayfish *Astacus leptodactylus* (63); CHH stimulated *in vitro* trans-epithelial electrical potential difference and Na^+^ influx in the posterior gills in the crab *Pachygrapsus marmoratus* (165) and was effective in restoring stress-induced decrease in hemolymph Na^+^ and K^+^ levels to the pre-stress levels in the freshwater crayfish *Cherax quadricarinatus* (85). High-affinity CHH binding sites were identified in, among other tested tissues, the gill of *C. maenas* and *in vitro* treatment of the gill with CHH significantly increased both cGMP and glucose levels in the tissue (86). In a study exemplifying the physiological roles of CHH at specific life-history stages, a gut-derived CHH, which was massively released into hemolymph during late pre-molt and ecdysis, was suggested to be involved in stimulating water and ion uptake in the crab *C. maenas*, causing body swelling for successful ecdysis and subsequent increase in animal size during post-molt (68). While these studies strongly implicate CHH as an important factor for iono/osmoreguation, the molecular target(s) on which CHH acts to achieve its regulatory activity were not directly addressed by these studies. It is however relevant to note that CHHs of the Christmas Island blue crab *Discoplax celeste*, which stimulated Na^+^ transport across the gill epithelia, had no effect on gill Na^+^/K^+^-ATPase or V-ATPase activity (75).

Characterization of the functional roles of CHH has led to the concept that CHH acts physiologically as a stress hormone (166). In various crustacean species, it has been shown that CHH mediates the stress-induced hyperglycemia in animals exposed to different stressors (extreme temperature, hypoxia, organic and inorganic pollutants, etc.), presumably metabolically acclimating animals to the stressful environment (50, 167–171). Interesting and insightful observations have been reported in studies working on crustaceans with distinct seasonal patterns of physiological demands (75, 76). The Christmas Island red crab (*Gecarcoidea natalis*) and blue crab (*D. celeste*) are two terrestrial brachyurans that undergo annual breeding migration towards the sea at the beginning of the wet season but remain inactive and fossorial during the dry season, and as such, were considered ideal species for investigating the roles played by CHH in metabolic and ionic homeostasis in an eco-physiological context as the endocrine status with respect to energy metabolism and osmoregulation should change seasonally (75, 76). One of the main findings derived from these studies is that in both species, while a 10-min extreme (forced) exercise stimulated a rapid and transient CHH release into the hemolymph followed by increases in hemolymph glucose levels, the hemolymph CHH levels in the migrating animals were actually significantly lower than those found in the animals during the dry season. This is in contrast to what would have been presumed based on the expected metabolic burden of the migration activity on the animals. It was further demonstrated that a negative feedback loop involving inhibition of CHH release by glucose existed only during the wet season but was uncoupled during the dry season. It was considered that the lower CHH levels in the wet season might be due to the inhibitory effect of high glucose levels, characteristic of migrating animals in the wet season when they continue to feed to conserve the glycogen stores that are to be utilized later for more strenuous activities, which deplete glycogen reserves upon the return migration. The significantly higher dry-season CHH levels, likely reflecting the lack of a functioning negative feedback loop at the time, might be metabolically advantageous when animals were fossorial with limited foraging activity (76). Alternatively, it was reasoned that the significantly higher CHH levels during the dry season, when conservation of water and ions would be crucial, might be closely related to the ionoregulatory activity of CHH (76). Indeed, experiments measuring branchial Na^+^ flux indicated that CHHs affect sodium uptake in the gill in a season-dependent manner attesting to the importance of CHH for ionic and water homeostasis (75, 76). Data generated from these studies highlight the intricate and intriguing aspects of CHH regulation that must be shaped in ways to meet the physiological demands characteristic of particular stages of the life history of the animals. These studies underline the importance of such functional approaches both in the field and in the laboratory for improving our understanding of the adaptive significance of CHH.

While CHH has been functionally characterized rather extensively, the functional role of CHH-L has been far less studied. It is however known that CHH-L, unlike CHH, does not have either hyperglycemic or ecdysteroidogenesis-inhibiting activity (47, 172, 173), and co-injection of CHH and CHH-L did not change the pattern of hyperglycemic responses in *C. maenas* injected with CHH alone. This rules out the possibility that CHH-L functions as a negative regulator of CHH (47). A recent study suggested that Pt-CHH2, a CHH-L peptide of the crab *Portunus trituberculatus*, could be involved in regulating gill Na^+^/K^+^-ATPase and carbonic anhydrase activity, since CHH dsRNA treatment decreased Pt-CHH2 transcript levels and significantly reduced the enzyme activity in the gills (60). Given that CHH is involved in osmotic regulation and that CHH, at least in the Christmas Island blue crab *D. celeste*, has no effect on gill Na^+^/K^+-^ATPase or V-ATPase activity (75), it is tempting to suggest that CHH and CHH-L peptides are both physiologically relevant factors for regulating water and ionic balance. However, these peptides likely do so by acting on distinct molecular targets, perhaps in a concerted manner.

Ion transport peptide was first identified based on its antidiuretic activity in the ileum in the desert locust *S. gregaria* (36–38). ITP, released from the corpora cardiaca, stimulates the ileum to transport Cl^−^ ion from lumen to hemolymph, driving water reabsorption. It is tempting to propose that ITP or ITP-L are involved in regulating processes critical for successful molting, as has been shown for CHH in crustaceans (68), but the evidence so far has not been conclusive [see (57)]. Studies using an RNAi method targeting expression of *itp*/*itp-I* transcripts in the red flour beetle *Tribolium castaneum* indicated that these peptides are important for adult eclosion, but less important for larval–larval or larval–pupal molting, based on observations of developmental defects and mortality (58). RNAi targeting expression of *itp*/*itp-l* in the brown planthopper *Nilaparvata Lugens* resulted in increased cuticle melanism and failed wing expansion (59). If and how the observed phenotypes during development are related to the water reabsorbing activity of ITP remain to be confirmed. In addition, because the phenotypes were mostly observed using dsRNA that simultaneously silenced *itp* and *itp-l*, the effects cannot be clearly assigned to individual peptides. Finally, functions of ITP are likely not limited to fluid reabsorption. In a recent study in *Drosophila melanogaster*, the roles of ITP have been extensively characterized as, not only enhancing water retention (estimated by the defecation rate), but also promoting thirst (estimated by the propensity to drink water and volume of water intake) and inhibiting intake of “dry food”, which are thought to work collectively to protect the animals from dehydration (174).

#### Pathogenesis and Immune Regulation (CHH)

There have also been interesting developments with regard to the pathophysiological roles of CHH. It has been shown in various crustacean species that a sub-lethal dose of lipopolysaccharide (LPS), a major component of the capsule of Gram-negative bacteria, elicited a significant hyperglycemic response (175), with a significant increase of hemolymph CHH levels as early as 30 min after treatment (80).

In another example in which CHH is implicated in a pathological condition, hemolymph glucose levels in the patently infected Norway lobster *Nephrops norvegicus*, parasitized by dinoflagellate *Hematodinium* sp., were dramatically decreased, indicating that the parasite, acting as a “carbohydrate sink”, was absorbing glucose from the hemolymph of the host, leading to a near depletion of the glycogen reserve in the hepatopancreas of the lobsters with late stages of patent infection (81). The observations that hemolymph CHH levels progressively increased as the severity of infection increased, while glycogen reserves were significantly decreasing, vividly highlight the glycogen-mobilizing effect of CHH. The opposite direction of changes in hemolymph glucose and CHH levels indicated that the negative feedback exerted by hemolymph glucose on CHH release is relieved in the patently infected hosts (81).

Similarly, in a study of white spot syndrome virus (WSSV) infection in *P. clarkii*, hemolymph CHH levels were significantly increased by WSSV. The virus-induced CHH release was rapid, commencing as early as 3 h post-infection, and long-lasting with the hemolymph CHH levels being significantly elevated for at least two days after the infection, leading to dramatic decreases in the CHH content in the eyestalk ganglia (79). However, hemolymph glucose levels in the infected hosts were not significantly higher than those in the uninfected animals, arguing for an enhanced glucose uptake by the host cells (79). Virus-induced alterations of metabolism, favoring viral replication and disease progression at the expenses of the host, have been reported in mammalian hosts [see (176)] and more recently also in crustaceans (*M. japonicus* and *Penaeus vannamei*) (177, 178). Thus, infection of WSSV alters the metabolism in the host cells, collectively known as the “invertebrate Warburg effect”, including a higher rate of glycolysis, the pentose phosphate pathway, ribonucleotide biosynthesis, glutaminolysis, and amino acid biosynthesis (177, 178), which are largely in accordance with the metabolic effects of CHH, as revealed by two metabolomics studies (77, 78). In fact, analysis of the muscle metabolome using an enrichment analysis showed that the “Warburg Effect”, among others, was significantly impacted when CHH expression was silenced (77). The combined data indicated that the virus-induced CHH release is at least partly responsible for inducing the Warburg effect (77–79). These studies illustrate how pathogens exploit the endocrine system of crustacean hosts, specifically increasing the CHH output and hence tilting the balance of host metabolism for the benefits of the pathogens. Silencing of *chh* gene expression was able to significantly decrease the viral load in the tissues and prolong the survival of the WSSV-infected *P. clarkii* (179).

Several reports studying the immunoregulatory effects of CHH in the Pacific white shrimp *L. vannamei* showed that recombinant CHH increased pathogen clearance and survival rates in pathogen-infected shrimps (82), and elevated total hemocyte count and the phagocytic activity of hemocyte (180). CHH treatment also affected the expression of several immune effector protein genes, including superoxide dismutase, LvRelish, and anti-microbial peptides, suggesting protective roles of CHH through modulating the immune activity of the shrimps (84, 180). It would be informative to investigate CHH-modulated immune responses under pathogenic conditions, which are invariantly characterized by significantly higher CHH levels as has been previously reported (79–81, 175).

CHH transcripts encoding CHH and related peptides, including CHH and CHH-L peptides and a novel truncated CHH, have been reported in hemocytes of *P. clarkii* (70, 72). Production of CHH peptides was also demonstrated, with a cell type-specific expression pattern, and CHH was able to stimulate guanylyl cyclase activity in the hemocyte membrane preparations (70). Wu et al. (70) suggested that the hemocyte-derived CHH may act on hemocytes in an autocrine/paracrine manner, regulating carbohydrate metabolism in crustacean hemocytes as they have been shown to be an important site for carbohydrate storage and metabolism (181–183), or have direct roles in regulating immune responses of the hemocyte.

#### Activities Overlapping With Those of Type-II Peptides (CHH)

CHH has been implicated in regulating other biological functions, including activities that functionally define the Type-II peptides. Thus in various species, CHH has been found to inhibit ecdysteroid synthesis (23, 65, 172, 184, 185), although its activity in this regard is less potent than MIH (65, 184, 185), as well as methyl farnesoate synthesis (186). The physiological significance of CHH-regulated ecdysteroidogenesis or methyl farnesoate synthesis has not yet been fully characterized. The functional overlap of CHH with the Type-II peptides may be the result of an evolutionary scenario in which the current CHHs (Type-I lineage) retain a certain degree of the functional pleiotropy of an ancestral peptide, while the Type-II lineage evolved towards peptides with more specialized functions (31).

### Type-II Peptides

#### Inhibition of Steroidogenesis in the Y-Organ by MIH

Eyestalk ablation has been known for a long time to shorten molt interval and induce molting in many crustacean species. Based on these observations, Zeleny (7) proposed the existence of a “molt-inhibiting” factor in the eyestalk. Follow-up studies in various species have repeatedly shown that eyestalk ablation leads to an increase in ecdysteroid production by the Y-organ and in hemolymph ecdysteroid levels (187–189). This factor has since been purified, sequenced, and named molt-inhibiting hormone (MIH) (20, 24, 184). Injection of recombinant MIH has been shown to prolong the inter-molt duration (190). Adding eyestalk extract or synthetic MIH to the incubation medium significantly diminished the secretion of ecdysteroids by the Y-organ *in vitro* (191, 192). Correspondingly, using ^125^I-MIH as the ligand, the existence of MIH-specific binding sites was detected in Y-organ membrane preparations from *C. maenas* (193), *Callinectes sapidus* (194), and *M*. *japonicus* (195). Altogether, these data strongly suggest that MIH acts directly on Y-organs to suppress ecdysteroidogenesis.

According to the above findings, a model has been proposed that the Y-organ ecdysteroidogenesis is suppressed by MIH during much of the molt cycle, only to be relieved from the suppression during the pre-molt stage, when MIH levels are low [see (196)]. Normally, hemolymph MIH levels are rather low (typically at the levels of fmol/ml) and difficult to quantify precisely. Available data regarding hemolymph MIH titers throughout the molt cycle are limited to three species, *C. maenas*, *C. sapidus*, and *P. clarkii* (197–199). Data in line with this hypothesis mainly come from the variation of MIH transcript levels throughout the molt cycle. In *C. sapidus*, *L. vannamei*, *M. nipponense*, and *Scylla paramamosain*, MIH transcript levels were relatively high during the inter-molt stage (stage C), declined gradually in the pre-molt stage (stage D), and then returned to a level similar to that of the inter-molt after molting (stage A/B) (111, 132, 200, 201). The hemolymph ecdysteroid levels in *C. sapidus* and *L. vannamei* displayed a corresponding elevation during pre-molt stage when *mih* gene expression was low (111, 200). Unexpectedly, a significant drop in hemolymph MIH levels was observed only in *P. clarkii* (197), whereas those in *C. maenas* and *C. sapidus* remained unchanged during pre-molt stage (198, 199), which contradict the proposed hypothesis. Thus, the model is considered to be incomplete, mainly because the absence of the pre-molt drop in MIH levels, at least in certain species, is not accounted for. In this regard, Chung and Webster first noted that the effect of MIH on the Y-organ of *C*. *maenas* is molt stage-dependent. The inhibitory effect of MIH on ecdysteroidogenesis was the highest (~60%) during inter-molt stage. The effect then dropped significantly during pre-molt and post-molt stages (less than 10%), indicating that the Y-organ became refractory to MIH stimuli at these stages (185). Similar observations were also reported in *P. clarkii* that MIH significantly inhibited ecdysteroidogenesis during inter-molt stage (~80%), and this inhibitory effect became much weaker (less than 10%) during the middle-premolt stage (192, 202). Pharmacological studies showed that the activity of phosphodiesterase 1 (PDE1), a calcium/calmodulin-activated PDE isoform, is closely related to the decreased responsiveness of the Y-organ to the inhibition by MIH during the pre-molt stage (192).

Regardless of the inconsistencies in the results obtained from different species, silencing of MIH expression utilizing MIH dsRNA resulted in a significant acceleration of molt frequencies (132, 203, 204) and elevation of ecdysteroids in the hemolymph (203), indicating a definite role of MIH in the regulation of crustacean molting and growth.

#### Stimulation of Vitellogenesis by MIH

Vitellogenin (Vg), the precursor of vitellin, is synthesized in the ovaries, hepatopancreas, or both (205–207). It is cleaved into smaller subunits in the hemolymph, and then stored as vitellin in growing oocytes (208, 209). Therefore, levels of *vg* transcripts in target tissues and Vg in hemolymph are commonly used as indicators of the progression of female reproduction in crustaceans (210). More recently, a novel function of MIH in stimulating ovarian growth *via* inducing vitellogenesis has been reported in a few species. In *C. sapidus*, MIH is capable of inducing hepatopancreatic *vg* mRNA expression and Vg secretion at mid-vitellogenesis stage (211). The hemolymph MIH titers in female crab were about four-times higher at the mid-vitellogenesis stage than those at the pre-vitellogenesis stage. Additionally, MIH-specific binding sites have been identified on hepatopancreas membrane preparations (194), indicating that MIH might act directly on the hepatopancreas to increase Vg production. Interestingly, the number of MIH binding sites on the hepatopancreas of adult females shows an “ovarian stage-dependent” variation that is two-times higher at the mid-vitellogenesis stage than at the pre-vitellogenesis or early vitellogenesis stage (194). Similar findings were also reported in *Metapenaeus ensis* and *L. vannamei* in that one of the two MIH isoforms of each species (namely, MeMIH-B and Liv-MIH2) stimulated vitellogenesis and promoted ovarian growth. *In vitro* incubation of ovarian explants and/or hepatopancreas with recombinant MIH resulted in the upregulation of *vg* transcript levels in *M. ensis* and *L. vannamei* (114, 212). Injection of recombinant MIH increased Vg content in both hemolymph and the ovary in *M. ensis* and induced ovarian maturation in *L. vannamei*. Additionally, injection of dsRNA also reduces MeMIH-B transcript levels in the hepatopancreas and ovary, resulting in lower Vg levels in the hemolymph. These combined data demonstrate a stimulatory role for MIH on female reproduction (114, 194, 211, 212). Regarding crabs with a reproductive phase accompanied by a terminal anecdysis, Zmora et al. (211) concluded that MIH is a vital regulator coordinating the reciprocal antagonism of molt and reproduction by keeping animals at an anecdysial status and stimulating vitellogenesis when the animals are sexually maturing.

#### Inhibition of Ovarian Maturation by GIH

GIH is another member in the Type-II group. After its first identification in *H. americanus* (25), GIH has been identified in several other species mainly through molecular cloning (213–216). Recombinant GIH decreased *in vitro vg* transcript levels in the ovary (216) and GIH dsRNA decreased *vg* transcript levels in the ovary of *P. monodon* and the hepatopancreas *of L. vannamei* (215, 216). A single injection of GIH dsRNA was capable of suppressing *gih* expression for at least 30 days, leading to ovarian maturation and spawning in *P. monodon* (217).

#### Inhibition of Methyl Farnesoate Synthesis by MOIH

Methyl farnesoate (MF), the sesquiterpenoid structurally related to insect juvenile hormone III (218), is a secretory product of the mandibular organ which participates in controlling growth and reproduction in crustaceans (219–222). The existence of an inhibitory factor that negatively regulates MF synthesis was proposed based on the observation that eyestalk ablation caused an increase in hemolymph MF levels in the hemolymph (223), while injection of eyestalk extract reversed the effect of eyestalk ablation (224).

In *C. pagurus*, two MOIHs (MOIH-1 and MOIH-2), which suppressed MF synthesis *in vitro*, have been purified from the sinus gland extract and characterized. MOIH-1 and MOIH-2, unblocked at both ends, exhibits significant sequence similarity with MIHs (26). Their gene structure and sequence similarity indicated that the *moih* and *mih* genes arose by divergence following a gene duplication event (118). An interesting issue regarding MOIH is the apparently restricted existence of MOIHs in the cancrid crabs (225), suggesting that the gene duplication event was relatively recent, probably not earlier than the divergence of the *Cancer* genus (118). In some non-cancrid brachyurans (*L. emarginata* and *C. maenas*), CHH inhibits MF synthesis (186, 226), probably assuming the role of MOIH, whereas in *C. pagurus*, functionally and structurally distinct CHH and MOIH are present (26). Whether *moih* genes are present and expressed in other brachyuran taxa is unknown.

## Peptide Structure, Signal Transduction Pathways, and Receptors

### Peptide Structure

The first structural model of the CHH superfamily peptide was that of MIH (Pej-MIH) from *M. japonicus* resolved by nuclear magnetic resonance (NMR) spectroscopy. It consists of a long N-terminal tail, followed by five alpha helices (α1−α5), and a C-terminal tail (92). Subsequently, two more structures of the crustacean member peptides have been elucidated: Pej-SGP-I-Gly, the glycine-extended precursor of a *M. japonicus* CHH by X-ray crystallography (91), and Sco-CHH-L, the CHH-L from the pericardial organ of *S. olivacea* by NMR spectroscopy (Protein Data Bank: 5XS1). Although topologically equivalent helices—α2, α3, and α4 (π4 in Sco-CHH-L)—are present in the core of the three structures, Pej-MIH and Sco-CHH-L are more topologically resemble one another than Pej-SGP-I-Gly (**Figure 3**). Thus, Pej-MIH and Sco-CHH-L additionally have a topologically similar C-terminal α5, which brings the C- and N-terminal ends sterically close each other (**Figure 3**). In contrast, Pej-SGP-I-Gly, which lacks an α5, has a relatively long α4 followed by a C-terminal tail that is kept away from the N-terminal end (**Figure 3**). Because of the limited availability of the C-terminal amidated Pej-CHH, Pej-SGP-I-Gly, a non-amidated precursor was used for structural determination (91). Post-translational amidation at the C-terminal increased the α-helical content of CHH (139, 140, 172), indicating that the C-terminal modification renders structural changes. Thus, while additional structures of the CHH-superfamily peptides (*e.g.*, amidated CHH and ITP) are needed for validation, it is likely that the folding pattern shared by Pej-MIH and Sco-CHH-L is a common theme for the crustacean and insect peptides of the superfamily. Functionally critical residues at the terminal regions of MIH, CHH, and ITP have been demonstrated using mutated recombinant peptides (87–90, 139, 140, 227). It is likely that parts of the structure, consisting of the sterically close C- and N-terminal regions of the peptide, where the functionally critical residues are located, play important roles in forming the binding site for receptor interaction and activation.

### Signal Transduction Pathways and Receptors

Radiolabeled peptides (125-I) have been used in several studies to identify the potential target tissues of CHH, MIH, or CHH-L and to profile the binding characteristics of the presumed receptor (86, 193, 228, 229). Generally, each of the crustacean members of the superfamily binds to a distinct binding site (receptor) with high specificity. In the shore crab *C. maenas*, Y-organs clearly have separate and highly specific binding sites for CHH and MIH (193). Similarly, in the blue crab *C. sapidus*, displacement experiments in multiple tissues also revealed that CHH and CHH-L peptide each has discrete specific binding sites (229). Another aspect of CHH binding specificity was illustrated by studies on hepatopancreas membranes from *C. maenas* and *O. limosus* (228). Membranes derived from *C. maenas* had a much lower affinity for *O. limosus* CHH than for *C. maenas* CHH (228), reflecting the species-specificity of CHH in terms of hyperglycemic activity (152).

A wealth of literature has been accumulated over the years regarding the signal transduction pathways of the CHH-superfamily peptides, mostly concerning MIH, CHH, and ITP. These include studies in various species utilizing *in vitro* or *in vivo* assays with tissue preparations or hormones, cyclic nucleotide analogues, and pharmacological agents inhibiting or stimulating activity of enzymes involved in the signaling pathways. In general, the studies have concluded that cyclic adenosine monophosphate (cAMP), cyclic guanosine monophosphate (cGMP), or both act as intracellular signaling messengers mediating the action of the CHH-superfamily peptides. However, experimental data are in several instances inconsistent and contradictory, particularly for studies involving MIH. Until recently, the nature (receptor guanylyl cyclase vs. adenylyl cyclase-activating G protein-coupled receptor) of the putative receptor for the CHH-superfamily peptides were only speculated upon based on data derived from the early studies. In a more recent study of the silkworm *B. mori*, orphan G protein-coupled receptors (GPCRs) were determined to be receptors for ITPs (ITP and ITP-L peptides), the characterization of which provided data for building a model of the signaling transduction pathway of ITPs (94), which is in general similar to an earlier one suggested for MIH (230).

### CHH and CHH-L

Early studies investigating CHH signal transduction pathways implicated both cyclic nucleotides as second messengers mediating the action of the hormone (156, 157). Thus, CHH preparations increased cGMP and cAMP levels *in vivo* in several target tissues, including the hepatopancreas and muscle of the crayfish, *O. limosus*. In *in vitro* incubations of the hepatopancreas, cGMP levels were significantly increased by CHH, which was followed by release of glucose into the tissue incubation media, whereas cAMP levels were already elevated during the incubation and not further increased by hormonal treatment (156). Further, injection of CHH into the eyestalk-ablated crayfish increased cGMP and cAMP levels and decreased glycogen synthase activity in the abdominal muscle. Cyclic nucleotide analogues mimicked the effect of CHH preparations in inhibiting synthase activity (157). In *M. japonicus*, bilateral eyestalk ablation decreased intracellular cGMP levels of the hepatopancreas, with little effect on cAMP levels; corroboratively, exposure of tissues to recombinant CHH significantly increased the levels of cGMP, but not those of cAMP (231). The CHH-induced increase in cGMP levels in the muscle of *H. americanus* (232) was due to stimulation of the membrane-bound guanylyl cyclase (mGC), as cGMP increased not only in intact tissue but also in the isolated membrane preparations (233). Involvement of soluble GC (sGC) in the CHH-induced cGMP increase was considered unlikely, as cytoplasmic sGC activity was not stimulated by CHH and the CHH-induced cGMP increase was not blocked by methylene blue, an inhibitor of the nitric oxide (NO)-activated sGC (233). These combined results favor a scenario in which CHH binds and activates an mGC, thus elevating intracellular levels of cGMP as a second messenger, while changes in cAMP, if present, could occur downstream to receptor activation and cGMP elevation. An mGC (PcGC-M2), which was identified by cDNA cloning in the muscle of *P. clarkii*, was found to contain the signature domains characteristic of receptor GC (rGC), including an extracellular ligand-binding domain, a single transmembrane domain, and intracellular kinase-like and cyclase catalytic domains (234). PcGC-M2 is widely expressed in several target tissues of CHH (234), although the binding ligand for PcGC-M2 has not yet been determined.

The signaling pathway activated by CHH-L is much less characterized. In the blue crab *C. sapidus*, CHH-L (pericardial organ-CHH) significantly increased cGMP production in several tissues including scaphognathites, heart, midgut, hindgut, and abdominal muscles (229).

### MIH

Results obtained from studies of the MIH-mediated suppression of ecdysteroidogenesis in the Y-organs in different species are often contradictory. On the one hand, cGMP was shown to be an important second messenger of MIH in several species, including *O. limosus*, *C, sapidus*, and *P. clarkii*. Thus, Y-organ incubated with native or recombinant MIH resulted in an increase of cGMP, but not cAMP, in these species (192, 202, 235). In related experiments, cGMP invariably played a crucial role in the mediation of MIH action: a cGMP analogue (8-Br-cGMP) significantly suppressed ecdysteroid production by Y-organ of *C. sapidus*, but neither cAMP analogues (db-cAMP or 8-Br-cAMP) nor an activator of adenylyl cyclase (forskolin) had a detectable effect on ecdysteroidogenesis (236); addition of synthetic MIH to the incubation medium increased cGMP levels, but not cAMP levels, in *P. clarkii* Y-organs (192). Corroborative observations were also reported for *C. maenas* Y-organ in which treatment with purified MIH produced a large and sustained increase in intracellular cGMP levels (237). These results are consistent with a model in which cGMP functions as a second messenger in the cellular action of MIH and that the receptor for MIH is likely a rGC. On the other hand, studies in *Cancer antennarius* showed that adding eyestalk extract (which contains a wide variety of bioactive compounds, including MIH) to incubation of Y-organs resulted in an increase in cAMP levels (189). Additionally, db-cAMP and agents (forskolin and choleragen) that increased intracellular cAMP, each mimicked the inhibitory action of MIH, while 3’,5’-cGMP did not. It was thus concluded that cAMP mediates MIH-induced suppression of ecdysteroid production (189).

Data supporting the notion that the MIH receptor is an mGC and that cGMP functions as a second messenger were obtained in follow-up studies in *C. sapidus*. Thus, a cDNA (*CsGC-YO1*) encoding an rGC was cloned from *C. sapidus* Y-organ (238). Immunohistochemical staining using a primary antibody raised against the extracellular domain showed that CsGC-YO1 is located on the Y-organ cell membrane. Y-organ preconditioned with the anti-extracellular domain antibody became refractory to the stimulation of MIH. In addition, sodium nitroprusside (an NO donor) failed to inhibit ecdysteroidogenesis in Y-organ of *C. sapidus*, suggesting that NO/sGC/cGMP signaling pathway is not involved in the MIH action (196). Finally, the transcript levels of *CsGC-YO1* in Y-organ of the inter-molt animals were four- and two-times higher than those in the pre-molt and post-molt Y-organs, respectively (239).

Alternatively, a model hypothesizing that the MIH receptor is a G protein-coupled receptor (GPCR) was proposed (230) based on data obtained mainly from the crab *Gecarcinus lateralis* (240–243). In combination with previous related studies (230, 243) and the transcriptomic analysis, the *G. lateralis* Y-organ was proposed to go through a four-stage transition (basal, activated, committed, and repressed stages) from inter-molt (stage C_4_), early pre-molt (stage D_0_), middle pre-molt (stage D_1_, D_2_) and late pre-molt stage (stage D_4_), respectively (244, 245). The MIH signaling pathway plays a dominant role in the inter-molt and early pre-molt stage to suppress ecdysteroid production. According to the model, ligand activation of MIH receptor leads to, *via* a G protein, stimulation of adenylyl cyclase and subsequent cAMP-protein kinase A (PKA) pathway. Phosphorylation of enzymes by PKA in turn causes inhibition of ecdysteroidogenesis. Another phosphorylation event activated by PKA leads to an increase in membrane calcium conductance, resulting in Ca^2+^ influx that activates NO-sensitive sGC pathways through Ca^2+^/calmodulin activation of nitric oxide synthase. Increase in cGMP levels and subsequent activation of protein kinase G ultimately lead to inhibition of ecdysteroidogenesis. This working hypothesis is sustained by a series of experimental studies. First, analysis of *G. lateralis* Y-organ transcriptome indicated the expression of signaling components mentioned above (including adenylyl cyclases, PKA, PKG, calmodulin, NOS, NO-sensitive guanylyl cyclase (GC-I), and GPCRs) (95, 121, 246). Second, the production of ecdysteroids *in vitro* was repressed by NO donors (SNAP and SE175) and by cAMP and cGMP analogues (247, 248). Third, an adenylyl cyclase (AC) activator (forskolin) inhibited the production of ecdysteroids (248). Fourth, in combination with IBMX, an sGC activator (YC-1) inhibited ecdysteroid secretion in Y-organ (247). Finally, eyestalk ablation led to the up-regulation of NO-independent-NOS, GC-I, and GC-III mRNA levels, causing the Y-organ to be more sensitive to MIH stimuli, presumably a compensatory response to the removal of MIH (240).

### GIH and MOIH

Information about GIH and MOIH signaling cascades are still limited. Effects of various pharmacological reagents have been tested in an *in vitro* assay using *vg* mRNA expression in ovarian explants from *M. japonicus* (249). Results showed that db-cAMP, db-cGMP, forskolin, and IBMX mimicked the inhibitory effects of GIH in reducing the levels of *vg* mRNA in a dose-dependent manner. Similar results were also obtained when A23187 (calcium ionophore) and PMA (an activator of protein kinase C) were added into the incubation medium. These results suggested that cyclic nucleotides, calcium, and protein kinase C are involved in regulating Vg transcription in the ovary (249). However, whether these signaling components were coupled to GIH activation has not been determined. Recently, a study on the mode of GIH action was carried out in *L. vannamei* using *vg* expression in the hepatopancreas as the bioassay. According to Chen et al. (250), *in vivo* injection and *in vitro* incubation of hepatopancreatic primary cells with recombinant GIH elevated the levels of intracellular cGMP, but not cAMP or nitric oxide, indicating that GIH exerts a cGMP-mediated inhibitory action. Pharmacological reagents were then tested to characterize the GIH signaling pathway. Results showed that GIH employs an rGC/cGMP/PKG signaling pathway in inhibiting *vg* expression; phosphorylation of c-Jun N-terminal kinases and upregulation of expression of p38MAPK (a mitogen-activated protein kinase) were implicated as signaling events downstream to PKG activation.

For MOIH, the only result has come from a study of *C. pagurus*, in which an increase in cAMP was observed with hormonal treatment and with cAMP analogues that mimicked the action of MOIH, implying cAMP as a second messenger for MOIH (251).

### ITP/ITP-L

**Figure 5:**
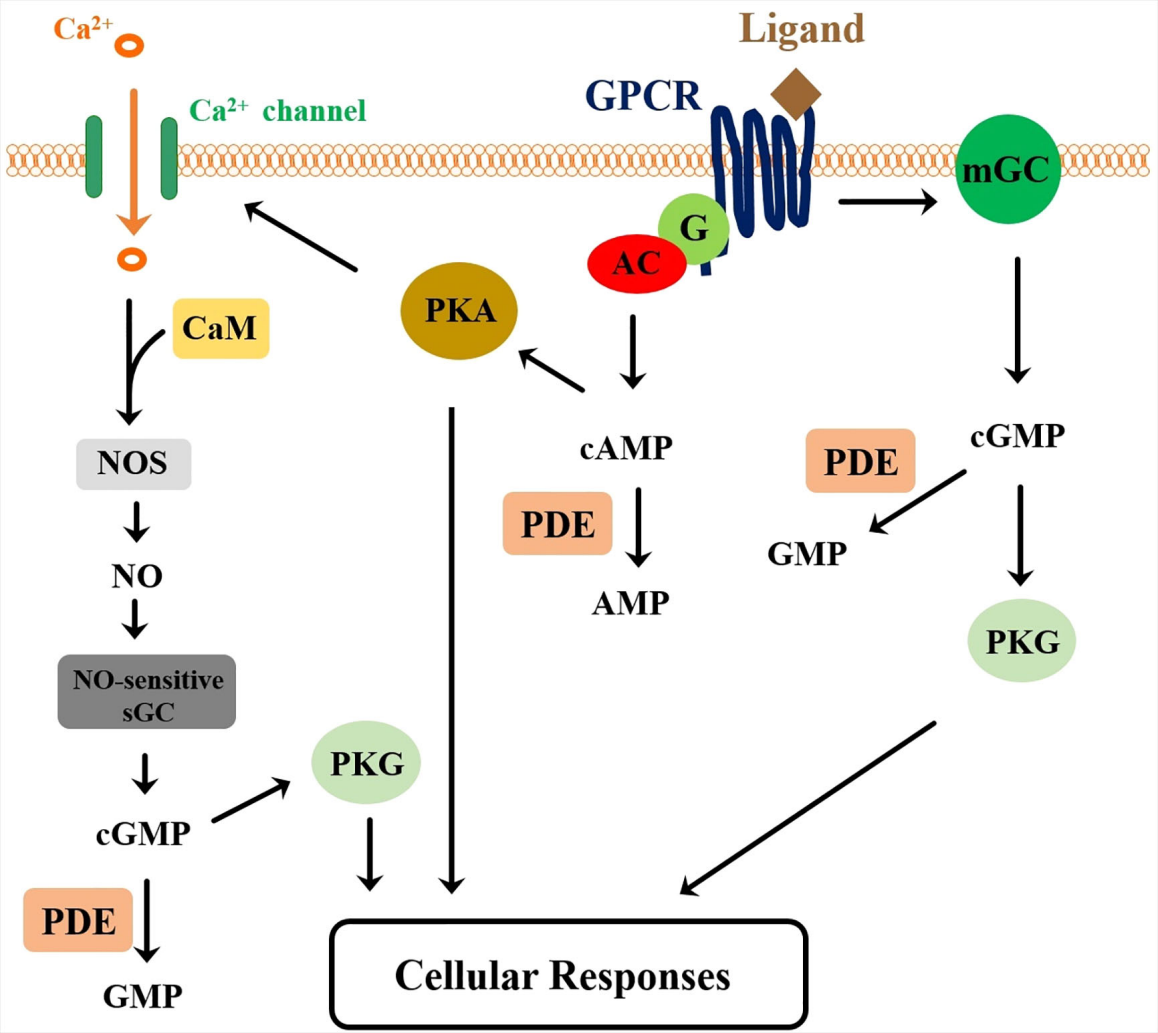
A proposed signaling pathway for the crustacean and insect member peptides of the CHH-superfamily. A G protein-coupled receptor (GPCR) functions as the receptor for the peptide ligands. Ligand-receptor interaction leads to activation of the adenylyl cyclase (AC), *via* a coupling G protein (G), and of the membrane guanylyl cyclase (mGC), resulting respectively in the elevation of cAMP and cGMP levels. Activation of protein kinase A (PKA) by cAMP leads to the increase of intracellular Ca^2+^ levels, through phosphorylation of membrane calcium channel. Ca^2+^/Calmodulin (CaM) complex stimulates nitric oxide synthase (NOS), increasing NO production, which in turn activates NO-sensitive soluble GC (sGC) for higher levels of cGMP production. Downstream events activated by the combined actions of PKA and PKG ultimately lead to cellular responses. Cyclic nucleotides are degraded by phosphodiesterases (PDEs). This model is a composite derived from Chang and Mykles (230) and Nagai et al. (94).

cAMP, cGMP, and agents that increase cAMP levels individually were able to mimic the effect of ITP on ileal short-circuit current. Additionally, synthetic ITP elevated intracellular levels of both cyclic nucleotides in a dose-dependent manner (36, 252), again implying the involvement of these two cyclic nucleotides in ITP signal transduction. Importantly, receptors of ITP and ITP-L peptides have recently been identified and characterized in the silkworm *B. mori* (94). *B. mori* orphan neuropeptide G protein-coupled receptors (BNGRs), obtained through *in silico* mining of the silkworm genome, were screened using a Ca^2+^-imaging for activation by recombinant ITP and ITP-L peptides in HEK293T cells co-expressing a BNGR and a promiscuous mouse G_α15_. Out of the 34 BNGRs tested, three Class-A BNGRs reacted positively to activation by ITPs, with BNGR-A2 and -A34 responding to ITP (EC_50_: 1.1 x 10^-8^ M and 1.3 x 10^-8^ M, respectively) and BNGR-A24 to ITP-L (EC_50_: 2.6 x 10^-8^ M). Interaction between ITPs and BNGRs was evaluated by an *in vitro* assay that showed co-localization of the ligand and BNGR at the cell membrane of CHO cells heterologously expressing the receptor; the interaction occurred in a ligand-receptor-specific manner, consistent with the data for the distinct receptor responses to ligand activation observed using Ca^2+^ imaging. Stimulation of *B. mori* ovary-derived (BmN) cells by either ITP or ITP-L significantly increased the intracellular cGMP levels. Coupling of the BNGRs to the ITPs-induced cGMP signaling was demonstrated by observations that simultaneous knockdown of *bngr-A2* and *-A34* significantly decreased the cGMP response to ITP in BmN cells, whereas knockdown of *bngr-A24* led to decreased cGMP responses to ITP-L; transient expression of *bngr-A24* potentiated the response of BmN cells to ITP-L. The involvement of mGC and sGC in the signaling of ITPs was examined in BmN cells using, respectively, dsRNA targeting BmGyc76c (a *B. mori* mGC) or ODQ (a selective and potent inhibitor of NO-sensitive sGC), showing that both GC forms are involved in signaling *via* ITPs through cGMP production (94). A model for the signaling pathway of ITPs in BmN cells was proposed (94), which is in general similar to the MIH model proposed by Chang and Mykles (230), except that it includes the involvement of both mGC and sGC (**Figure 5**). Thus, activation of BNGR receptors by their respective ligands leads to activation of adenylyl cyclase *via* G-protein coupling and to activation of mGC. Like the MIH model, one of the events downstream to activation of protein kinase A activation is the increase in membrane calcium conductance, leading to Ca^2+^ influx, which in turn stimulates NO-sensitive sGC by activating Ca^2+^-calmodulin-dependent NO synthase. Increases in cGMP levels, due to the actions of both mGC and sGC, then activates protein kinase G, which together with protein kinase A, result in cellular responses (**Figure 5**).

Identification of receptor for insect ITPs not only represents an important breakthrough for insect studies but also provides an opportunity to identify the receptors for crustacean member peptides. Previous studies of molecular mass of the CHH- and MIH-binding proteins, respectively, in the hepatopancreas and Y-organ of *C. sapidus* and *M. japonicus* showed that the estimated values (51–70 kDa) were within the molecular mass range of typical GPCRs (194, 195). Given the concept that the peptide ligands co-evolve with their receptors, phylogenetic analysis of the crustacean and insect GPCRs has been performed searching for the crustacean orthologs that are phylogenetically clustered with the receptor for insect ITPs. Thus, analysis of transcriptome data derived from *P. clarkii* tissues has uncovered GPCRs, *Procambarus* GPCRs A52, A53 and A63, that clustered with BNGR-A34, and *Procambarus* GPCR A9 with BNGR-A24, with A52 and A63 being abundantly expressed in the hepatopancreas, implying these GPCRs could be the crayfish CHH, MIH, or CHH-L receptor (97). In addition, analysis of the data derived from the spiny lobster *Sagmariasus verreauxi* revealed 2 annotated GPCRs (Sv-GPCRA11 and 12) that are phylogenetically clustered with BNGR-A34 (96). In the blackback land crab *G. lateralis* Y organ transcriptome 3 GPCRs (Gl-GPCRA9, Gl-GPCRA10, Gl-GPCRA12) were similarly identified using phylogenetic analysis as potential CHH-like receptors, as these sequences clustered into the putative CHH receptors clade (95). Expression of one of the GPCRs, Gl-GPCRA12, decreased in late pre-molt and post-molt stages, suggesting that it may be the MIH receptor (95). These results are promising and provide receptor candidates for testing of ligand binding and receptor activation to truly establishing the status of these receptors for the crustacean peptides.

The signaling model based on experimental data mainly derived from the studies of *G. lateralis* and *B. mori* (94, 230) is attracting and probably applicable to the crustacean and insect members of the CHH superfamily (**Figure 5**). Several major coupling events however need experimental verification, including coupling of GPCR activation to adenylyl cyclase, GPCR activation of membrane GC (as suggested in the ITPs signaling), PKA phosphorylation-induced Ca^2+^ influx and subsequent NO production. In addition, for the model to be applicable to the crustacean CHH-superfamily peptides, including CHH, the model would have to be refined, based on data from additional studies, to accommodate the apparently contradictory data derived from other studies. For example, in response to hormonal stimulation, the change in cAMP levels was usually small and insignificant (192, 235, 237) and manipulation of cAMP levels or adenylyl cyclase activity did not mimic the effect of hormonal treatment (192). Further, it is interesting note that the lobster CHH stimulated the guanylyl cyclase activity in isolated membrane (233). Thus, GPCR activation of mGC would occur within the membrane without signaling through the cytoplasm. A GPCR-activated mGC event within the membranes, if proven, would probably represent a novel mode of GPCR signaling mechanism. Finally, it is now feasible to conduct experiments for ligand-receptor interaction and activation, with the availability of identified receptors and of information regarding ligand structure and structure-function relationship of several peptides, including MIH, CHH, CHH-L, ITP (87–93).

## Conclusion and Prospective Developments

The field of study of the CHH superfamily is now at a challenging and promising phase. With more research efforts, most likely through mining newly available genomes and transcriptomes from additional ecdysozoan taxa, it is certain that new members of the superfamily will be discovered. The phylogenetic profile would be updated as new members are admitted to the superfamily. The fact remains that the study of crustaceans suffers, at least at the level of the malacostracans, and in comparison with hexapods, from genomes of often enormous size, which constitutes an obstacle in technical as well as financial terms. The hypothetical multiplication of genomes of these taxa has certainly actively promoted diversity with the appearance of paralogues and processes of sub-functionalization.

Evolutionary recruitment of the CHH-superfamily peptides as venom toxins (43) elegantly illustrates how changes in the structural characteristics of the peptides during the course of evolution has led to the emergence of novel functions, which indeed carries significant implications for the structure-function relationships of the crustacean member peptides. In this context, we could also question the fact that, in crustaceans where isoforms of the superfamily members are the most numerous, no venomous forms have yet been identified (except for the remipede *Speleonectes tulumensis* (253) that has toxins unrelated to the CHH superfamily), whereas this is the case in other major arthropod phyla. On the other hand, post-transcriptional and post-translational mechanisms that are well known for structurally and functionally diversifying the superfamily peptides are expected (and have actually been suggested by recent studies) to be also working on the venom peptide-encoding genes and the peptides. We likely will see more instances where results gained from the study of one group of peptides of the superfamily “complement” or “echo” those from studies of others.

While the crustacean and insect member peptides of the CHH superfamily have been characterized and identified, each with a distinct functional assay, progress in the functional front has been limited, if compared to the number of publications devoted to identification of the peptides and genes. Technical resources are essential for functional studies. Recombinant protein production, RNAi, and various functional genomics methods are now readily available and have indeed been widely used by researchers in the field. In this context, one particular technical obstacle for the study of CHH and ITP is the limited availability of biologically active peptides, production of which usually relies on costly and tedious modifications (*e.g.*, the C-terminal amidation) and refolding of bacterially-produced peptides. The obstacle becomes almost prohibitively challenging for structural studies that require large amounts of peptide. Alternatives, *e.g.*, chemical synthesis (133, 254) and eukaryotic expression systems (88, 255), could be explored to optimize the production of biologically active peptides at larger scales. Advancements in the functional front are expected if more research efforts are to be made. A persuasive example is a sophisticated model for the regulation of steroidogenesis in the Y-organ built mainly based upon functional genomics data [see (245)]. Additional genetic resources enjoyed by the study of insects, especially those working in the fruit fly *D. melanogaster*, as exemplified by a study of ITP (174), would probably render a faster development for the study of insect member peptides. On the application side, initial attempts were made in developing methods for manipulation of ovarian maturation and control of viral diseases respectively through silencing of *gih* and *chh* genes by RNAi (179, 217), a technical approach that has been put to practical use in aquaculture industry in the case of manipulating a crustacean insulin-like androgenic gland hormone (256).

Functional studies would be greatly aided by the identification of receptors for the superfamily peptides, which was finally realized by the discovery of the receptors for the silkworm ITPs (94). Crustacean orthologues of the silkworm ITP receptors were suggested to be the receptors for the crustacean member peptides (95–97). The pressing issue would be to provide experimental data testing whether the ligands bind and activate the candidate receptors. With the receptor for the peptides being identified, confirmation of the target tissues of a given peptide and the signal transduction pathways coupled to receptor activation could be examined. Interesting and physiologically relevant questions could be asked, for instance, regarding a function shared by two superfamily peptides (functional overlap) or a multi-functional (pleiotropic) peptide. For example, whether the Y-organ cells express both MIH and CHH receptors, as suggested by the radiolabeled-ligand binding experiments? How are the respective signaling pathways activated by MIH and CHH orchestrated in regulating steroidogenesis in the Y-organ? The prospective developments for the functional study of the CHH superfamily in a post-receptor era are promising and the discoveries to be made will certainly be rewarding!

## Author Contributions

Writing: H-YC, J-YT, and C-YL. Coordination: C-YL.

## Conflict of Interest

The authors declare that the research was conducted in the absence of any commercial or financial relationships that could be construed as a potential conflict of interest.
